# Navigating youth, smartphones, and policy: a balanced perspective on digital wellbeing

**DOI:** 10.1038/s41390-025-04288-3

**Published:** 2025-07-29

**Authors:** Selina McCoy, Ann Marcus-Quinn

**Affiliations:** 1 Social Research, Economic and Social Research Institute, Dublin, Ireland; 2 Department of Sociology, Trinity College Dublin, Dublin, Ireland; 3 School of English, Irish and Communication, University of Limerick, Limerick, Ireland

## Abstract

Smartphone use among young people is framed as a post-pandemic concern, with increased screen time and online distractions attributed to lockdown-induced dependency. However, this narrative overlooks the trajectory of digital policy in education where education departments globally were encouraging the integration of student-owned devices into classroom learning before the pandemic. The growing focus on links between the digital lives of children and adolescents and their mental wellbeing are often based on correlative data with few longitudinal or experimental studies; there is a notable absence of studies which successfully disentangle the effects of different types of smartphone/social media usag—separating learning/educational from recreational uses. However, a growing number of governments have introduced restrictive policies to limit access. This one-size-fits-all approach may inadvertently exacerbate inequalities or ignore the social realities of young people’s lives. While regulatory efforts may form part of a broader digital policy toolkit, bans alone are insufficient. They do little to address the underlying needs that drive youth engagement with smartphones; the human desire for social connection, access to information, and autonomy. A more effective response must be holistic: combining regulation with education, digital literacy, and the co-creation of safe digital spaces that support both protection and participation.

## Introduction

This paper reflects on debates around smartphones, social media and digital activities in shaping young people’s wellbeing and satisfaction. In current discourse, smartphone use among young people is often framed as a post-pandemic concern, with increased screen time and online distractions frequently attributed to lockdown-induced dependency. However, this narrative overlooks the historical trajectory of digital policy in education. A 2013 report by the European Commission collected and benchmarked information from 31 European countries on the access, use, competence and attitudes of students and teachers regarding ICT in schools. It found that between 28% and 46% of students reported using their personal mobile phones for learning purposes at school at least once a week. Irrespective of whether it was officially permitted, students were increasingly bringing their own devices into the classroom and using them as educational tools.^1^ Prior to the COVID-19 pandemic, departments of education globally were actively encouraging the integration of student-owned devices into classroom learning. As early as 2017, policy instruments such as circulars and directives laid important groundwork for the informal incorporation of smartphones into educational settings, particularly in resource-constrained schools without access to 1:1 laptop or tablet schemes.

While many countries’ digital strategies for schools emphasised pedagogy over hardware, in practice implementation often relied on devices students already possessed - commonly smartphones. This disconnect between strategic policy aims and their practical enactment highlights what has been identified as a persistent tension in policymaking: the gap between reactive responses to immediate needs and the absence of a longer-term strategic framework.^2^ In this light, the increasing presence of smartphones in schools is less a by-product of the pandemic and more a foreseeable consequence of earlier policy directions that were, in many cases, reactive and technologically permissive.

## The evidence—what do we really know?

Adolescents around the world have experienced a decline in their mental health, with their digital lives frequently cited as underlying these trends.^3^ Drawing on cohort data in Germany, one study illustrates increasingly problematic use of smartphones and their association with the overall declining quality of life of children over the last seven years.^4^ But the links between the digital lives of children and adolescents and their mental wellbeing are often based on correlative data; few studies take more rigorous approaches, using longitudinal or experimental evidence, and fewer still clearly support the notion that there is a causal relationship between smartphones and social media use, and mental wellbeing.^5^ Further, researchers have largely overlooked how the kind of instruments used to measure mental health, as well as the populations being studied, limits their ability to draw clear conclusions.^6^ The need to focus on individual differences is key, with gender, for example, particularly important in understanding the impacts.^7,8^ Overall, there is a notable absence of studies which successfully disentangle the effects of different types of smartphone/social media usage—separating learning/educational from recreational uses. To better understand the complex effects shaping young people in the digital age, more transparent collaborative research between independent researchers and the technology industry, and granular user-engagement data, is needed.^9^

## Policy reactions—global responses and their limits

In recent years, a growing number of governments have introduced restrictive policies aimed at limiting young people’s use of smartphones and social media, citing concerns over mental health, as well as distraction, and online safety. Sweden, for example, has moved towards banning smartphones in primary classrooms, aligning with broader efforts to refocus on traditional forms of learning. In 2024 the Australian Government passed a new law, the Online Safety Amendment (Social Media Minimum Age) Bill 2024. This law, to take effect by December 2025, has attracted international attention and introduces a mandatory minimum age of 16 for accounts on some social media platforms. Controversially, parents cannot give their consent to let under-16s use these platforms, marking a new departure in terms of parent rights. At a surface level such actions offer clear, decisive action in the face of complex technological challenges, and resonate with public anxieties about youth well-being. However, such bans are fraught with practical, human rights and ethical implications. Age verification systems can be circumvented, and bans also risk pushing young users onto less regulated platforms or creating generational disconnects in digital literacy. The Australian Human Rights Commission has indicated the legislation is potentially in breach of the UN Convention on the Rights of the Child, the International Covenant on Civil and Political Rights (ICCPR) and International Covenant on Economic, Social and Cultural Rights (ICESCR).^10^

Mobile devices and social media can serve as essential tools for safety, communication, and family coordination, especially for older adolescents needing greater independence. UNICEF Australia is working to capture young people’s reactions to the recent social media ban in schools across the country.^11^ By amplifying their voices, the organisation aims to understand how this policy impacts their daily lives and online experiences, while encouraging youth to express their views on the broader implications for their digital rights and well-being. Ultimately, a one-size-fits-all approach may inadvertently exacerbate inequalities or ignore the social realities of young people’s lives.

## A way forward—constructive, balanced digital citizenship

Reflecting public sentiment, there are many organisations, and individuals, actively campaigning for government intervention on the issue of smartphone use among young people. In the UK, a Suffolk mother began *Smartphone Free Childhood*, which became a national campaign to ban smartphones for children under 14. In Spain, *Adolescence Free of Mobile Phones*, again initiated by parents, is campaigning for no smartphones for under 16 s. In Ireland, *Genfree* is working to raise awareness within school communities about the potential negative effects that smartphone access can have on brain development and mental health. Through their initiative, they encourage parents to sign a petition and provide a template for emailing government representatives, urging them to take action.

Parents are also increasingly interested in smartphones which offer a solution to manage screen time and limit access to distracting apps. The Balance Phone is one such solution, offering a simplified interface with a focus on essential functions such as communication (e.g., messaging apps like WhatsApp), entertainment (e.g., Spotify), and education (e.g., Duolingo, Kindle). The aim is to provide a healthier balance between digital connectivity and personal well-being by reducing the overwhelming range of apps and social media platforms that can negatively impact mental health, focus, and overall development.

Voluntary collective agreements among parents of primary school children to delay children’s access to smartphones are also growing. The “No Smartphone” initiatives seen in parts of Ireland, represent a promising grassroots approach to addressing concerns around digital overexposure, cyberbullying, and mental health. These agreements, which rely on community-wide parental cooperation, help reduce peer pressure and create a more uniform standard that benefits all children. Crucially, for such interventions to be most effective, they must be implemented before the teenage years, ideally during primary school, when habits and digital behaviours are still forming. Early implementation not only delays premature exposure to potentially harmful online environments but also lays the groundwork for more responsible and mindful smartphone use in adolescence, supported by ongoing digital literacy education and parental involvement.

While regulatory efforts may form part of a broader digital policy toolkit, bans alone are insufficient. They do little to address the underlying needs that drive youth engagement with smartphones; the human desire for social connection, access to information, and autonomy. A more effective response must be holistic: combining regulation with education, digital literacy, and the co-creation of safe digital spaces that support both protection and participation. Such an approach is evident in the *European strategy for a better internet for kids*, which seeks to ensure that children are protected, respected, and empowered online. It centres on three pillars, Safe Digital Experiences—protecting children from harmful and illegal online content, conduct, and risks; Digital Empowerment—enabling all children to acquire the necessary skills and competences to make sound choices and express themselves safely and responsibly online; and Active Participation—respecting children by giving them a say in the digital environment, with more child-led activities to foster innovative and creative safe digital experiences.^12^

## Concluding remarks

Rather than relying solely on restrictive measures, governments need to invest in education-based initiatives that empower young people and their families to navigate the digital world safely. For example, Ireland’s Webwise, an initiative of the Department of Education co-funded by the European Commission, offers comprehensive resources for digital literacy and online safety. Similarly, Safer Internet Day, held annually in over 180 countries, presents a valuable opportunity to promote responsible online behaviour through schools and community engagement. Acceptable Use Policies (AUPs), widely implemented across educational contexts, also present significant potential as instruments for guiding responsible smartphone and social media engagement among students. Reflections from school leaders underscore the view that clearly articulated and consistently implemented AUPs may serve a pivotal role in advancing digital wellbeing within school communities.^13^ Of course, this does not absolve technology companies from their responsibilities. It is crucial to implement stronger regulations for these companies, including setting quality standards for children’s content, controlling data extraction and algorithms, and enforcing stricter privacy laws.^10^ Technology is a constant aspect of contemporary life, and it is continuously evolving. Policymakers must adopt a flexible approach that maximises its benefits while safeguarding young people from potential risks.
